# Fracture of the Clavicle

**Published:** 1876-11

**Authors:** J. C. Watson

**Affiliations:** Saltville, Ala.


### FRACTURE OF THE CLAVICLE.

By J. C. WATSON, M.D., Saltville, Ala.

A simple mode of treating fracture of clavicle is to have made two loosely-fitting rings of any stout material, well stuffed with cotton or wool (the latter better), and slipping one over each arm, well on to the shoulder, pass a piece of stout, inelastic material through the rings behind; and now you have a brace which can be pulled upon, and the shoulder securely fixed, without the least danger of slipping. The ring in the axilla of the fractured side can be fashioned and stuffed to answer a better purpose, I think, than the wedge-shaped of the Fox apparatus; for being a part of the ring, it cannot slip from the axilla, as is sometimes the case with the Fox pad.

Now the brace behind I think an improvement on the figure of 8 bandage, as it can be tightened at will, and what is of more importance, there is no chaffing of the axilla and the body, from the encircling of the roller bandage. Now give a sling to elevate the shoulder, and a few turns of roller to fix the elbow to the side, and the apparatus is complete, carrying the shoulder upward, outward, and backward, and maintaining the parts securely and comfortably in the desired position.

This simple plan I have used with success for twenty years, and its mention may be of interest to some inexperienced brother.

